# From Inpatient to Clinic to Home to Hospice and Back: Using the “Pop Up” Pediatric Palliative Model of Care

**DOI:** 10.3390/children5050055

**Published:** 2018-04-26

**Authors:** Martha F. Mherekumombe

**Affiliations:** Department of Palliative Care, The Children’s Hospital at Westmead, The Sydney Children’s Hospitals Network, Westmead, NSW 2145, Australia; martha.mherekumombe@health.nsw.gov.au

**Keywords:** pediatric, palliative, Pop Up, discharge, inpatient, life-limiting, hospice, hospital, goals of care

## Abstract

Children and young people with life-limiting illnesses who need palliative care often have complex diverse medical conditions that may involve multiple hospital presentations, medical admissions, care, or transfer to other medical care facilities. In order to provide patients with holistic care in any location, palliative care clinicians need to carefully consider the ways to maintain continuity of care which enhances the child’s quality of life. An emerging model of care known as “Pop Up” describes the approaches to supporting children and young people in any facility. A Pop Up is a specific intervention over and above the care that is provided to a child, young person and their family aimed at improving the confidence of local care providers to deliver ongoing care. This paper looks at some of the factors related to care transfer for pediatric palliative patients from one care facility to another, home and the impact of this on the family and medical care.

## 1. Introduction

Children and young people with life-limiting illnesses requiring palliative care spend a variable amount of time in the hospital. They have variable care requirements related to their underlying disease or condition, and at some point of their medical treatment, their care needs may need to continue in another care facility. Whatever the reason for such a transfer, it is important to provide the best support for the child and the caregivers in the preferred or appropriate location of care where possible. Parents and caregivers often prefer being closer to home to maintain some family normality [1,2,3].

These diverse and often complex care needs should be considered when arranging patient transfer along with an assessment of the local health care’s capacity to care for the child and their family. This paper discusses the general factors related to the patient transfer of care, maintaining holistic care, and the impact of the transfer on the child and family. 

## 2. Case Report

Patrick (not his actual name) was an eight-month-old baby with a neurodegenerative disorder. He was the youngest child born to non-consanguineous Caucasian parents Sam and Melissa (not actual names). Melissa had an uncomplicated pregnancy and Patrick was born at term with a normal birth weight at a primary care facility. Around 30 h of age, he was noted to have respiratory distress and hypothermia and was transferred to the neonatal care unit. Shortly after admission, he developed seizures and investigations revealed a deranged pathology suggestive of a severe metabolic disorder, after which he was transferred to a tertiary hospital for specialist treatment.

Over the next few weeks, his stay was complicated by periods of instability. After a month his condition stabilized and he was discharged home. His time at home was very difficult and exhausting for the family. At the age of 5 months, Patrick’s parents were given the choice to consider a liver transplant and after much deliberation, his parents made the difficult decision not to proceed and instead opted to maximize his quality of life and a referral to palliative care was made.

Patrick’s seizures continued to be a concern and he deteriorated further necessitating a readmission to the tertiary hospital. Patrick’s goals of care were determined, and these were to limit life-prolonging interventions that had little benefit and for Patrick to be cared for closer to home.

Patrick was in the hospital for a further month. When his condition stabilized plans were made for a transfer to his local healthcare facility as a step down prior to the final discharge home. A multidisciplinary meeting was convened to review his medical and nursing needs. Changes were made to Patrick’s care plan to ensure local services were able to support him closer to home. This included revising his medications and limiting technology such as equipment devices that were unable to be used by the local health providers.

### 2.1. The Local Healthcare Facility

The transfer was uneventful; unfortunately, after a short period of stability Patrick’s clinical condition continued to deteriorate and he was admitted to the local hospital with the care plan that had been put in place. He remained at the facility for three weeks before being discharged home with ongoing support re-initiated by the local community health facility and the community palliative care service.

### 2.2. Hospital

Patrick enjoyed six weeks at home and was re-presented to the hospital with further deterioration. Though the goals of care were clear, Patrick’s parents needed further reassurance that there was no reversible disorder contributing to the deterioration. This admission was different because Patrick was dying. He was transferred to the hospice as per his parents’ wishes, where he died peacefully in his mother’s arms.

Reflecting back the family reported that being at the local healthcare facility enabled them to spend time away from the tertiary hospital. The goals of care for Patrick as he deteriorated were for him to be cared for at home or as close to home as possible. His advance care plan was to ensure comfort by managing symptoms, offering psychosocial and spiritual support. They were comforted by the knowledge that they did the best they could for their little boy. Being at the hospice for Patrick’s death was like a home to them, this meant they could be parents to both the children and feel “normal” for a very short time. 

## 3. Transfer from an Acute Care Facility

The hallmark of pediatric palliative care is providing holistic care. This can be achieved wherever a patient is located, and the care can vary globally. Patients are transferred between care facilities for various reasons [4]. It is important to consider the patient, their family’s wishes, and their preferences during a transfer from an acute care facility to ensure that their quality of life is maintained. Goals of care form the framework in which we define what is important for the family by aligning the needs and interests of the child with the goals and wishes of the family [5]. During the planning processes and transfer, these goals of care frame the basis of support.

Goals of care may change during a child’s illness and should be re-assessed throughout episodes of a life-threatening condition, clinical deterioration, or transfer of clinical care [6,7]. At the time of clinical diagnosis or referral to palliative care, it is important to establish the family’s overarching wishes, to document these, and to inform the relevant agencies. 

The process of planning a transfer from an acute facility to a non-acute facility can be multifaceted, involving considerations around care complexity, technology, and equipment support [4]. Other reported pertinent elements include the functional and social aspects of the child and family, including the psychosocial and spiritual domains [8]. Involving key healthcare providers in coordinating the discharge ensures that the patient has the required supports in place, the necessary paperwork such as a current discharge summary, and an acute management plan for deterioration along with advanced care directives [4,9]. Other considerations are finances, insurance, and local governance processes such as policies and procedures related to the transfer or discharge to ensure that there is a continuity of care and to minimize unnecessary transfers [4]. 

A patient transfer occurs when a child is discharged from one facility to another. There are generally three described processes that are required to initiate discharge of a hospitalized child. These include specifying discharge goals, assessment of discharge healthcare needs, and identifying factors that could affect the child’s well-being [10]. Discharge readiness is a concept that signifies the ability to understand and execute the intended discharge care plan in a safe and timely manner [10].

Occasionally, to facilitate care transfer or discharge, the accepting provider may need to be supported to care for the child or young person and this support is given through adopting a model of care known as “Pop Up” [11]. 

### 3.1. Pop Up Model of Care

Pop Up model of care is a concept established to enhance access to specialist pediatric palliative care services when needed. The intervention facilitates timely and well-coordinated palliative care by providing in-time training and education to local health providers.

Through a Pop Up intervention, the specialist pediatric palliative care (SPPC) service builds the capacity of the local care providers in response to a clinical or family need. The SPPC team is a team of health professionals with formal training in the provision of pediatric palliative care. A Pop Up intervention occurs when an SPPC service responds to the needs of an individual child, young person, or family and builds capacity with the local community to establish a ‘bespoke’ pediatric network incorporating the following triad:

1. Family/caregiver.
2. Local health services (such as the family practitioner, community nursing, local hospital services, or local care facility).
3. Specialist pediatric palliative care (PPC) Service [11].

Central to a Pop Up is providing just-in-time training that aims to build capability and capacity for local health providers to continue delivering high-quality palliative care to the child and their family. Pop Up can also be used to support bereavement provision [11]. With this model of care, patients and families can advocate for the appropriate care within their goals of care which was important for Patrick’s parents. Patrick’s Pop Up was a combination of a face to face meeting and telemedicine which has been reported as an effective tool to provide consultations [12].

In the case study above, discharge planning from hospital commenced in the weeks prior to the transfer when Patrick was stable. The planning meetings helped to identify the level of support that was needed to maintain his quality of life in a non-tertiary setting. A Pop Up was convened closer to discharge and a management plan was outlined considering the local health provider capabilities and resources. Although Patrick’s parents wanted him to be cared for in a non-tertiary facility and to be at home, the representations to the hospital were related to their anxiety. This anxiety related to the increased burden of care. Parents and caregivers often experience this when caring for a child with a life-limiting illness as the child or young person’s condition deteriorates. Patrick’s parents received the appropriate support and counseling to assist them with their grief and the impending loss of their son. Local supports were notified to intensify their support during this time. Children with life-limiting illnesses are reported as having frequent readmissions and are at risk for recurrent hospitalizations, and this needs to be anticipated [13]. In preparing for discharge, care planning to ensure that the necessary supports and an assessment of the family’s home is important. The role of palliative care includes helping to decrease hospitalizations and the unmet care needs when the child leaves the hospital [13].

A comprehensive management plan was formulated during Patrick’s discharge planning, with the contact details of the main medical providers, pertinent medical and social history, medications, and an emergency medical care plan. The plan was formalized during the Pop Up and it included plans of end-of-life care. The hospice was the family’s preferred location for the end-of-life care and all these plans were finalized with all the relevant parties including the receiving team with a predetermined transfer date. In some countries, there are inpatient facilities known as hospices which are care facilities providing support, respite, and end-of-life care for children with life-limiting illnesses and their families. These facilities can also be used when children need to transition to a less acute facility from a hospital, prior to being discharged home. The term hospice in some other countries refers to a model of care providing end-of-life care and is not inferred as such in this section, but rather the former [14,15,16]. 

There are reported benefits to Pop Up, which include:

1. Enabling discussions around the current and anticipated medical concerns in a considerate and sensitive manner.
2. Assessing all aspects pertaining to care such as equipment, provisions, or needs.
3. Individualizing the comprehensive care plan to the child’s needs with the local services.
4. Providing networking opportunities between the hospital and community health providers.
5. Providing family-centered care and supporting family goals including choices for location of the care.
6. Providing SPPC support to manage the escalating symptoms in a timely manner [11].

### 3.2. Challenges Relating to Care Transfer

There are few challenges in a patient transfer that can occur and these may include the discontinuity of care and changes to treatment including medication regimes and therapies [4]. Discontinuity of care is often an outcome of poor communication related to the swift transfer with inadequate preparation, limited finance, conflict, limited medical staffing, or inexperienced staff at the recipient facility [5]. The transfer processes can be complex, contingent on the healthcare systems and the family working together. Other factors include the inclusion of the appropriate health professionals, completing discharge requirements, having post discharge contingency plans, and providing education to patients and parents [4,9]. 

Components of hospital discharge have also been examined and aspects such as the medical care team’s involvement, addressing clinical needs, contingency plans, and parent readiness rank highly in relative importance among clinicians [4,9]. Pediatric palliative care services and primary care teams need to work collaboratively to provide continuity of care and maintain the patient’s and families’ well-being [2,17]. Continuity of care is only possible when realistic expectations are in place. It is also helpful to have one contact person such as the pediatrician or a senior nurse. Parents and caregivers need to be prepared for the transfer process as much as possible by providing them with information about the new facility and the relevant updated actions or emergency management plans. The handover processes should occur between physicians and also among the nursing staff. Information pertaining to advanced care planning should also be communicated and this informs the focus of care and helps establish and communicate the care expectations.

Other factors to consider which affect the transfer includes aspects relating to the child’s health and safety such as having the appropriate medical insurance, finances, and parent-health care professional communication [10,12].

### 3.3. Impact on the Child/Family

Pediatric palliative care patients including those with rare medical disorders are becoming more challenging because their prognosis has changed with the advancement of medical technology and these children are living longer than they were previously. This uncertainty impacts decision making processes, families, and discharge or transfer plans [5]. The complex care needs of some of these patients are further complicated by having multiple specialties involved in patient care which can result in fragmentation and adversely affect the children [5]. Having a written plan for difficult symptoms is important because poor symptom management impacts negatively on the child and the family [18]. From the limited literature available, we know that the lives of patients can be disrupted during periods of transfer [12,19,20]. Further research on improving the patient experience is needed. 

## 4. Conclusions

Care planning is vital to the success of the transfer of care. Pop Up was used to facilitate the transfer in the case reported. The benefits of this have been reported, however, the processes for patient transfer from the hospital to their home or to another facility require further research. The impact of transfers on children and their families also needs to be explored to determine ways to improve patient care and patient experience. Some pertinent elements required for patient transfer has been discussed and each patient is an individual and needs to be considered on a case by case basis.
